# Book Reviews

**Published:** 1807-07

**Authors:** 


### CRITICAL ANALYSIS

OF THE

### RECENT PUBLICATIONS

ON THE

### DIFFERENT BRANCHES OF PHYSIC, SURGERY, AND MEDICAL PHILOSOPHY.

---

*An Enquiry into the Seat and Nature of Fever, deducible from the Phænomena, Causes, and Consequences of the Disease, the Effects of Remedies, and the Appearances on Dissection. In Two Parts. Part the First, containing the general Doctrine of Fever.* By HENRY CLUTTERBUCK, M. D. Member of the Royal College of Physicians, London, 8vo. 1807.

IT is very justly observed by the author, that since medicine has been reduced to something like fair induction from obvious facts, no one has attempted to define the absolute nature or seat of fever. His own attempts he urges are, to his best judgment, a fair and legitimate deduction from generally admitted facts, for the truth of which, he has appealed to the history of the disease, as delivered down to us from the most accredited writers and practitioners of all ages. It is strongly supported also by analogy, and is in unison, he conceives, with the general laws of the animal economy.

The work is divided into two parts. The first comprehends the general doctrine of fever, according to the principles assumed by the author. The second, which is still unpublished, will contain its particular application to the various states of the disease, with a more minute examination of the effect of remedies.

The preface concludes with a few very just observations, on the importance of adjusting remedies to symptoms, and on the injury many have suffered by the indiscriminate use of cordials in fevers.

The first chapter of the volume before us, contains some preliminary considerations on the laws of the system of health, on the nature of disease generally, and on the division into universal and local. Under the first head, after some general remarks on the complicated nature of the human body, and the difficulty of ascertaining the proximate causes of its actions, some useful observations are offered on the various susceptibilities peculiar to different parts; the effects of agents, [various applications] on different parts; on the same parts at different times, and on the relation or sympathy which exists between different parts, with the various causes to which they may be ascribed. Under the second head, it is suggested that the vascular system, sanguiferous and absorbent, is the great seat of disease. Some remarks on the laws of disease follow, particularly on the manner in which they supersede sede each other, on the division of symptoms, and on the means of distinguishing primary from secondary symptoms, and the uncertainty of inferences which may sometimes be drawn from even morbid dissections. Lastly, the author endeavours to prove that there is no such thing as an universal disease; that debility is never the immediate cause of disease.——This passage contains many useful remarks, referring principally to a doctrine at one time popular, and which we still find by some, if not defended, at least applauded. For want of room, we can only transcribe the following note.

"I shall take this opportunity of remarking, that the doctrine which supposes almost all diseases to be universal, and the whole system to be acting in a similar manner, either in excess or the reverse, owes any popularity it may have chanced to possess, more to its apparent simplicity, than to its consonance with truth and the laws of the animal œconomy. In diarrhœa and dysentery, which occupy a conspicuous place in the catalogue of *asthenic* diseases, the part affected and the general system are often in the opposite state of action, in regard to one another. The system is weak;—the intestines have all their actions preternaturally increased. This is seen in their augmented sensibility and consequent pain; in the excessive secretions poured out into their cavity; and in their increased peristaltic motion: these surely cannot be consequences of local debility or a weaker action of the parts. The same contrast might be shown to exist in a great number of other diseases, between the general system and the seat of topical affection."

There may seem a little obscurity in this passage itself. If there is no such thing as universal disease, it may be asked, what are those general diseases which are to be distinguished from local? But the author's intention is to show, that local disease may produce a general wrong action; that therefore we are to look for organic or local affection in our treatment of most diseases, though the symptoms may be general.

In the next chapter, Dr. C. attempts to show that fever is never to be considered as a disease of the whole system, but that "the brain is always the true seat of morbid affection in fever, and the source of all the symptoms which essentially belong to it, and which serve to distinguish it from other diseases." A remark follows, that this theory is confined to the idiopathic fevers of authors, unconnected with that general affection from topical inflammation, usually termed symptomatic.

The phænomena of fever are next considered, as indicating its seat. In attempting this, our author very properly refers to the authority of other writers of respectability, who must have been unbiassed by the conclusions he wishes to draw. By extracts from G. Fordyce, Huxham, Lind, Dewer, and De Mertens, he shows that fever, be the remote cause and the consequent symptoms varied in any other form, is always distinguishable by altered action about the the head. This section concludes with some ingenious remarks, to prove, that though the brain exerts more or less influence in every part of the system, yet that the actions of the heart, arteries, and other organs immediately concerned in the support of life, are much less dependant on that viscus, than those functions which connect man with the external world, which raise him above the scale of vegetable existence to the rank of an intellectual and free agent.

Two sections follow, showing that those animal functions, which the author derives principally from the brain, are more early affected than those vital functions which are less influenced by that organ. The latter, he conceives, are only affected in a secondary manner. The natural functions, viz. secretions, excretions, digestion, &c. are also said to be affected in a secondary manner, by the influence the brain is found to have on the organs subservient to those offices. Lastly, the other phenomena of fever, particularly hæmorrhages, petechiæ, vibices, &c. are all assigned to the same deficiency in the functions of the brain; and it is shown that such deficiency, on many occasions, induces that atony on the extremities of the vessels from which all these consequences may be traced.

The remote causes of fever are next traced; and after showing the insufficiency of the Cullenian doctrine, in deriving them all from human effluvia and marsh miasma, the author, from a general view of the subject, supported by various authorities, concludes, that though irritations, bodily as well as mental, may induce fever, yet that the brain is in all affected before actual fever is formed. This chapter closes with an account of the predisposing causes to, and consequences of fever. The first, it is shown, are principally connected with the mind, and the latter always attended with an impaired state of the intellectual faculties.

The 3d chapter on the nature of febrile action, is introduced by a summary of the former doctrine.

"No one will deny that, in fevers, the functions of the brain are greatly deranged, and that many of the most formidable symptoms of the disease may be referred directly to this source. I have given my reasons above for believing that the affection of the powers of sensation, thought, and voluntary motion, so remarkable in fevers, is not merely an accidental or casual occurrence, but essential to, and characteristic of, the disease; that it exists, in greater or less degree, in every case of idiopathic fever, while other parts of the system, that are less immediately subjected to the influence of the brain, as the organs of the vital and natural functions, are by no means necessarily or constantly deranged in fever; and that, when they are so, the derangement is neither uniform in kind, nor at all proportioned to the violence and danger of the disease.

"I shall next proceed to shew, that the disorder of the brain which takes place in fever is either a state of actual inflammation, or, or, at least, a condition nearly allied to it, as it contains the most essential characters of this affection. This will appear alike probable, whether we consider the phenomena of the disease, the causes, or the effects of remedies; and we shall afterwards see, that the opinion derives all the support from the dissection of bodies dead of fever, that could reasonably have been expected."

In pursuing this inquiry, the author begins with showing the analogy between fever and inflammation; and from the various symptoms, appearances on dissection, and authorities of the best writers, infers the probability that fever is only inflammation of the brain. We regret much that our limits will not permit us to be as particular as we could wish on this most important part of the work, but we must either transcribe the whole, or satisfy ourselves with a mere epitome; for throughout the whole, the author has paid as much attention to brevity as correctness. We shall, however, take some notice of the concluding part of the chapter, as most easily divested of the train of reasoning.

After showing the errors of Hippocrates, Erasistratus, Asclepiades, Themison, Galen; of Avicenna, and the Arabian School in general; of the chemical extravagancies of Paracelsus; the Archæus of Van Helmont; the mechanical theories of Borelli, Willis and others; the spasms of Hoffman, afterwards adopted with some little variation by Cullen, the lentors of Boerhaave.—"Of the hypotheses now mentioned, some are merely conjectural, founded on the supposition of a state of the system which has never been demonstrated to exist. Of this description are the *error loci* of Erasistratus, the *corpuscularian* doctrine of Asclepiades, the *humoural* doctrines of Galen and his followers, the *chemical* principles of Paracelsus and succeeding chemists, the *lentor* and *obstruction* of the mechanical sect, the *laxity* of Cole, &c. Others take up some one prominent and conspicuous symptom, and consider it as constituting the primary and essential part of the disease; such as the excess of heat, the profuse secretion of bile, the spasmodic constriction of the skin, or the debility or rather inability for muscular exertion; all of which are effects or consequences only, and not primary links in the chain of phenomena of fever."

The opinions concerning the local seat of fever are next stated: and after the mention of those modern authors who have acknowledged their ignorance of the subject, yet admitted the early affection of the brain, Dr. Home's interesting case of himself, is transcribed from "Medical Facts." On this the author, with great justice, remarks, that if such a physician could admit, that under inflammation of the brain, he felt all the symptoms usually attributed to low fever, the probability is, that they are the same disease; and this is confirmed by the history of an epidemic low fever, described by Dr. Home, which prevailed among the British troops in Flanders. In all these cases, the first symptoms were dulness of the intellect, which increased in many till till the disease ended fatally. The patients were not bled, on account of the general marks of debility. Only the bodies of two were opened, and the brain of only one, which was found in a state of suppuration.

"Here, (says our author) is an instance of epidemic fever, of the *low kind*, exhibiting after death unequivocal marks of previous inflammation in the brain. Bnt what were the symptoms in this individual case?—Were they such as to distinguish it from others of the same epidemic, indicating in a peculiar manner the presence of topical inflammation of the brain, according to the ordinary characters laid down of this disease?—By no means. "The patient had been," Dr. Home says, "in one of these slow fevers for a month; and was first seized with a vomiting and purging, which yielded to a vomit and injections. He lingered in this fever, sometimes complaining of a small pain in his head, till he was sent to the hospital, where, continuing two days in a low way, he was seized with slight convulsions, and expired."—In no case of fever, probably, could there be less reason to look for disorganization of the brain, than in the one before us. No raving, nor violent pain in the head, no redness of the eyes, not even delirium, is mentioned among the symptoms.

"Dr. Home, in his comment on this case, remarks, "that if we are to judge by all the symptoms, the brain was the principal seat of disease; but we dare not conclude, "says he," that every brain was affected in this manner. In this case, we see the substance of the brain converted into pus (and that too of no short standing, since the sinuses were so many), without any sudden or pressing symptoms. What shall we say," he adds, "of matter formed in the cerebellum, where the least disorder has hitherto been looked on as mortal? It overturns the doctrine of the schools."—It certainly does so, and not only with regard to this particular point, but to the whole doctrine of fever. It proves, with others, that inflammation and suppuration may take place in the brain, with scarcely any of the symptoms commonly assigned to phrenitis; without any, in short, but the mildest symptoms of fever. From the event of this case, it is probable that, had other dissections been made, similar appearances might have presented themselves. Yet we are not at liberty to infer, for reasons already stated, that such would have been generally, or even frequently, the case. When, however, we consider how rarely, in comparison with the whole of fevers, the state of the braiu has been examined after death; and how often, in the examinations which have been made, decided marks of inflammation have been perceived, a strong ground of suspicion, at least, is afforded, that the inflammation here was not merely an accidental occurrence, but the primary cause of the febrile symptoms, or rather the disease itself."

A short chapter follows on the diagnosis of fever, which the author has no difficulty of showing, never fails to be attended with affections of the sensorial functions. This is admitted by all authors, authors, and is said to be the only invariable symptom from which the following conclusion is drawn.

" In conformity, then, with the view of fever above given, we should consider it as a topical affection of the brain, founded in inflammation; in a word, as a variety of *phrenitis*, the essential characters of which it contains. The term *phrenitis*, however, is objectionable, as expressive of delirium or alienation of mind, which, though a very frequent, is not a necessary nor constant attendant on fever. The term *encephalitis*, as implying merely inflammation of the contents of the cranium, seems more appropriate, and is sufficiently comprehensive to embrace every variety of the disease.

" Must not fever, therefore, in future, be removed from the class of *universal* diseases (if there be any such), and ranked with the PHLEGMASIÆ, or topical inflammations, of nosologists? Like these, its characters are to be sought in the condition and feelings of the part affected, and in the state of its peculiar functions. Thus, in every proper fever, we shall find, in addition to the ordinary febrile symptoms of hot skin, irritated circulation, foulness of tongue, thirst, and deficient or irregular secretions,—pain in the head, generally of the throbbing kind, and extending along the continuation of the brain that is lodged in the channel of the spine; increased heat of the head, easily perceived on compressing it with the hands, even though the body and extremities be cold; unusual throbbing of the arteries in the neck and temples, suffusion of the eyes, and an altered expression of features easily to be perceived, but difficult to be described; together with disturbance of all the functions immediately belonging to the brain, as the *voluntary* and *mental* powers (both of which are always greatly weakened), and *sensation*, which, at different times and in different stages of the disease, is subject to be exalted, depressed, or otherwise depraved. If to these be added, irregularity in regard to sleep and watching, which, though common to many other diseases, belongs in a peculiar manner to the state of fever, we shall have characters always sufficient to enable us to detect the presence of fever in the system, affording at the same time the clearest indications of its nature and seat."

The last chapter, in the present volume, is on the various remedies for fevers, as they refer to the preceding doctrine.

If any thing can reconcile us more satisfactorily to the boldness of our author's theory, it is certainly the decisive authorities he produces in favour of early blood-letting. Though these are pretty generally known, and though it is scarcely necessary to mention any other names than Rush and Robert Jackson; yet it certainly does not redound much to the credit of the present practitioners, that two such names, so well supported, have been so little attended to. The only difficulty that stands in our author's way, is the decision of Dr. Fordyce, against the practice. We admire Dr. C's candour, we are unwilling to call it partiality, on this occasion; but we we are much at a loss to know what attention he can pay to the practical remarks on fever, of one who determines so pointedly the constant inutility, and sometimes absolute fatality, of early blood-letting in fevers. Allowing that Dr. F's practice was principally confined to London, the same may, we apprehend, be said of Dr. Clutterbuck's, and instances on record were equally open to both; they, however, may have been multiplied since the decease of the former.

We have given the spirit of this work with as much accuracy as our limits will admit, and we should feel ourselves very ungrateful were we not to acknowledge the obligations we are under to the author. Whether we consider his patient industry in the innumerable authorities he has produced, the satisfactory manner in which he has turned them to the support of his doctrine, or the candour with which he meets every difficulty and objection, we must admit, that we rarely meet with a question of so much importance treated with so much judgment, or with so regular a process of inductions from well founded facts. What adds much to Dr. C's merit, is the boldness with which he attacks a popular doctrine, and maintains one which leads to a practice always condemned, if unsuccessful, and too often undervalued, when attended with a most desirable issue.

As the present volume contains only one part of the work, it is with pleasure that we give the author an opportunity of explaining himself in some particulars, which to us are not satisfactory.

We cannot help wishing that the work had commenced, if not with a precise definition, at least with some definition of inflammation. That the brain is invariably affected in fevers cannot be questioned, and the stomach also. In short, *vires imminutæ* are in every part indisputable, excepting those of the absorbent vessels, which Dr. Clutterbuck has not failed to remark, are increased. But whilst we admit that the actions of the brain are altered, we are not perfectly satisfied that genuine inflammation always takes place. Even if we admit with the Author, that increased action is sufficient to constitute inflammation, we shall still be under the necessity of dividing inflammation, as Mr. Hunter has done, into different species, according to their sensible properties; and this is the more necessary, as Dr. Clutterbuck had admitted, that different, and even contradictory remedies, may be required under different states of inflammation in the brain.

"Topical inflammations, (we are told) are thus not only often relieved by increasing the actions of the system generally, but by stimulating applications to the part itself. These, by exciting the actions of the part still further, and perhaps also by diminishing at the same time its excitability with regard to the ordinary and healthful stimuli, seem, as it were, to induce fatigue in it; and, when the application is withdrawn, the action falls below that which is essential to inflammation, and approaches that of health. This is well illustrated in the case of inflammation produced by burns burns and scalds, which, in many cases, appears to be as successfully treated by stimulants of the most active kind, and even by heat itself, as by the application of cooling remedies, or of actual cold."

We cannot easily see the coincidence of the two facts here placed in illustration of each other. In scalds and burns, it is very certain that the parts recover themselves, however contradictory the treatment may be; but in most cases, we suspect that the applications have only a temporary effect; perhaps, in some instances they may interrupt, and in others facilitate the curative processes of Nature. But if topical inflammations, which have become chronic or habitual, are to be removed by stimulating remedies, we should impute such cures to the checking of an *error loci* of the ancient writers, or the superseding one action by another, according to the Hunterian theory. Or if we call every increased action inflammation, it will, as was just remarked, be absolutely necessary to define, as far as possible, the nature of the inflammation, since our practice must be regulated by it.

We should remark that every new action, however salutary, is attended with something like inflammation. If a slough is to be separated, the process is first an increased number and volume of the sanguiferous vessels. If suppuration is to take place, it is ushered in by the same process. Even ulceration is found invariably preceded by inflammation, as the previous blush on the integuments shows. In all such cases some increased action is necessary for the new process, and it is the business of art to prevent that action from being carried too far. In fever, however partial the disease may be, it must be admitted, that ultimately almost every part is affected; that is, that an almost universal change of action takes place. Not only the functions of the brain, but of the stomach, the skin, and often of the kidnies, are altered. That the head is very early affected cannot be doubted, and when this affection proceeds no further than to hebetate the senses, or to render the mind insensible to all external objects, this is considered by all physicians as a most desirable event. In fever, therefore, as a new series of actions is to take place in so important an organ, the attention of the physician should always be directed to that organ; and the least symptom of action being increased, so as to endanger its structure, should be watched with the most anxious care. If the cause which induces the fever suddenly invades a person in high health, the danger must be proportionate; and if the body has been from any cause previously heated, we perfectly agree with our author and those practitioners who urge early bleeding as a most important remedy.

Till we see the rest of this performance we are unwilling to say more. Perhaps we are already premature in the objections we have offered. However this may be, we cannot help congratulating the medical student on the acquisition of so valuable an addition to our stock of knowledge, on a disease which requires, on all occasions, casions, a promptness of decision, only to be acquired by practice, or by the support of that authority which is derived from such works as the present.

---

### *Observations on Morbid Poisons, Chronic and Acute, &c.* By Joseph Adams, M. D. &c.

( Concluded from Vol. xvii. pp. 567—574.)

On the subject of Sivvens our author is very minute. Besides an exact description of seven cases, which came under his own observation, in different stages, we have the accounts of most of the writers on the subject. From an examination of the ancients, particularly Celsus, as well as those surgeons of the middle ages, whose writings have been produced to prove the antiquity of the venereal disease, it seems satisfactorily proved, that sivvens is one of those local morbid poisons which has occasionally appeared, though never to such an extent as in a country where the custom has prevailed of smoaking with one common pipe. In the subsequent chapter, Yaws is described with not less minuteness. These are the only two diseases that have hitherto received a name, and can be confounded with the venereal. The peculiar marks by which they may be distinguished are carefully pointed out, after which the important subject of the anomalous Morbid Poisons is commenced.

This chapter comprehends a very enlarged view of such diseases as have been confounded with Syphilis; by which it appears, that the descriptions are all of them either deficient, or when sufficiently minute, that they are not applicable to the true venereal character. All the cases which yielded to acids are shown to come under one of these descriptions. Mr. Abernethy's " Account of Diseases resembling Syphilis" are next examined, and each is referred to its respective class, according to the arrangement in Dr. Adams's former edition of Morbid Poisons. This part of the work is highly important, and we regret much, that our limits will only admit a transcript of the conclusion which is, comprised in the form of Aphorisms.

" Whenever we see a sore in these parts without pain, and scarcely distinguishable from mere excoriations, we should content ourselves with the most simple applications, and without any internal remedies.

" If the sore heals firmly for some days, or if it continues stationary, or spreads only superficially ,without pain, we may be satisfied it is not venereal, or that it has not yet acquired a venereal character.

" If attended with pain, we may suspect a morbid poison of some kind.

" If the inflammation is considerable, and the disposition to ulcerate rapid, or slough should have commenced, we shall probably have fever at the same time. In all these cases we must attend only to the general and local symptoms, as in ordinary cases, by allaying the inflammation and fever. " If

"If the fever and ulceration both continue, our prognosis must be unfavorable; but the longer we delay the use of mercury, the greater will be the probability of success from it.

"If the disease is not soon relieved by mercury, we have reason to fear it will be exasperated by it; and if we find this the case, we must refer to those remedies which have been before suggested.

"If slough should have commenced, and its extent appear to be considerable, the probability is, that as soon as it is cast off, the part will skin over without granulation.

"If granulations follow the rapid separation of a slough, we must consider the case as common mortification.

"But it may have arisen from inflammation, excited by the presence of a morbid poison; we must, therefore, carefully examine whether any ulcer remains where the slough has not taken place, and watch its progress, so as to ascertain its character.

"If the slough is superficial, and the part from which it is separated looks particularly clean, that is, retains the crude surface of separation, neither skinning nor granulating, we may expect a succession of sloughs along the surface; and in the early stage of such a disease we shall gain nothing by mercury in any form.

"If, as the inflammation subsides, or after the slough is separated, we find, instead of healing, a hard and somewhat painful ulcer, without any restoration of parts, we may be certain of a chancre.—In these cases the sloughs will generally be small, almost circular, and about the size of a beginning chancre.

"If the ulceration should be slow, and without the character of chancre, the fever somewhat abating, we have every reason to believe the disease will cease spontaneously, or that as soon as the constitution is become familiar with it, that it will yield to mercury.

"This, in addition to what has been said of the soft wart and the thick lipped ulcer, will, I trust, be sufficient for every practical direction in the treatment of primary anomalous symptoms.

"But whilst there is this certainty in distinguishing primary, it must be admitted that secondary symptoms are by no means so readily ascertained. It is but justice to add, that these were the only cases in which Mr Abernethy was undecided; and we cannot be too grateful for the number of instances he has produced, and the descriptions he has given, imperfect as some of them may be.

"The difficulty of distinguishing secondary symptoms, seems to arise from our associating so much the idea of copper spots in the skin, with the secondary symptoms of syphilis. We should recollect that all eruptions, whose inflammation is slight, and whose progress is not rapid, must begin in this manner. Even in the exanthemata, if we watch carefully, we shall discover something of this kind as the eruption commences. The appearance arises only from the transparency of the cuticle, which exposes the

 slightly slightly inflamed cutis underneath. In those morbid poisons, which have not a certain course to run, the progress is so slow, that this copper spot is more circumscribed and permament. It is of different complexions, according to the degree of inflammation, which in syphilis is so trifling, as at first only to produce a slight separation of the cuticle, which continues to be formed again for some time, before ulceration or even scabbing takes place. The same happens in many other morbid poisons; nor ought we to be more surprised, if we cannot distinguish these eruptions at first, than at our incapacity, in their early stages, to distinguish small-pox from chicken-pox."

A chapter follows on Leprosy. In this the various diseases, at different times, denominated Elephantiasis or Leprosy, are accurately distinguished. In the description of what our author calls the Elephantiasis of the ancients, or the Arabian Leprosy, we must not omit the notice of one very striking particular. Most writers describe the afflicted as peculiarly salacious, but this is so far from the case, that after a careful examination of all the subjects in a Lazar House in the island of Madeira, our author establishes it as a general law, that "when the disease attacks a male subject before he arrives at the age of puberty, he never acquires that state; and such as are attacked later in life, gradually lose the power of procreation as far as can be judged by the changes which take place in their organs." This chapter concludes with some general remarks concerning the source of that confusion of terms, Elephantiasis and Leprosy, which may be traced in profane and sacred writers.

The last chapter of this part of the work is confined to the controversy, whether the Itch arises from an insect or not. The conclusion is, that a disease somewhat resembling Itch is the effect of an insect, a figure of which is given; but that in the true Itch, no one has, hitherto, been able to discover any thing animated.

The second part is on "Morbid Poisons attended with Fever." The first chapter on the various causes of fever, contains some short remarks on the injury this branch of pathology has sustained by the adoption of the term Typhus, which has for some time been applied to every kind of low fever. The author's definition of fever follows, and the various causes which induce such a change in the actions of the economy are enumerated. The origin of Typhus fever among the poor during severe winters or seasons of scarcity, leads to a consideration of those affections of the mind, which induce similar effects; a short passage of which we shall transcribe.

"Thanks to an improved state of society these national calamities are rare, which is the more consolatory, as the remedy is not within the power of those who have the nearest view of the evil. Let us then all be cautious how we contribute to others within our own scope. Let us remember, that these are only among the impressions which impede the common actions of life, and which must, if continued, produce local or constitutional disease.

Above

Above all, let us reflect that these causes will be proportionate to the susceptibility of the subject to whom they are applied. The different periods of life render us susceptible to different impressions, which are varied according to the nature of our education or rank in life, permanent or transitory from the same causes, but most of all from the original dispositions with which we are born. The child is distressed if less praised than his equals; the youth if he feels conscious that his progress is slower; but most of all the man just rising to settled life, if his prospects are clouded, his character suspected, or his inclinations opposed in such a manner, that he is forced to forego what he conceives absolutely necessary to his future happiness. Under any of these circumstances the apprehension of disappointing his friends—that contracted view he has of life, which induces him to suppose that the eyes of all are fixed upon him, and the keen sensibility peculiar to his age, produce a conflict that has often proved fatal to the most ingenuous minds. From having seen this period so frequently fatal to young physicians, it is often suspected to arise from contagion. This may sometimes be the case; but those who converse with the commercial world on the subject, will find that the event is by no means uncommon in men of very different pursuits at that particular age.

"In the other sex these causes operate still more powerfully, in proportion as the constitutional irritability is greater, the view of external objects more contracted, and the necessity of preserving an unsullied reputation the more imposing. But in both it is much to be wished we could recollect, that the best are the principal sufferers. In both there are characters who would die at the suspicion of an act, which others would be proud of concealing, and almost indifferent to if discovered. A severe expression, to which a sense of duty prevented a retort, an imputation to which pride prevented a reply, have been considered as proofs of tame submission or guilt, and the repetition or want of explanation have proved fatal to the best of minds.

"'Tis the brain of the victim that tempers the dart!"

"We ought further to remark, that the various changes the constitution undergoes at different periods, though for the most part so gradual as to be but little observed, are sometimes attended with those difficulties which will end in fever. This is more noticed in females, but I doubt whether the change in men is not often more striking. At what is called the more advanced climacteric, a long continued hectic ends in a continued, though slow, fever, the issue of which depends much on external circumstances, and from which, if the patient recovers, he is afterwards congratulated by his friends on a renovation of health and youth.

"I have mentioned these among the causes of fever, which are continually occurring, some of which are, from their nature, too often unknown by the physician, and too little attended to by mankind at large. When some of these causes occur a in circle, whose habitation is rarely enlivened by the full rays of the sun—when it occurs at a season during which, those who have the happiness of a blazing hearth, are distressed at an open crevice, physicians well know, and have often deplored, the consequence. In large towns infectious air is the most common cause of fever among the poor, and the most alarming to the rich, as it is impossible to say by what means they may be exposed to it. But whilst we are thus cautious, let us not increase the exercise of that selfishness, which, where life is concerned, is too apt to predominate. Let us reflect, that, as these causes have been more accurately explored, they have been found less formidable, and that prudent courage consists in ascertaining a danger, pusilanimity in flying from it. The love of life, inseparably connected with its posession, has sometimes unnecessarily superseded all those charities, which to man can only render life valuable: or the severity of state policy has confined innocent victims to a charnel-house, from which they might have escaped with security to themselves, and without danger to their persecutors."

The second chapter is " on the Manner in which different Fevers supersede each other. Of the infectious Atmosphere, endemic effluvia, and pestilential Constitutions of the Air." The title of this chapter gives some idea of its importance, but the perusal enhances it greatly. Under the " infectious atmosphere," are comprehended those diseases which originate in cottages, poor houses, prisons, camps, or fleets; the endemic effluvia are confined to marsh miasmata or the filth of ill-paved towns, during a season of hot and calm weather; the pestilential constitutions of the air, are those epidemics which spread over a considerable portion of the globe. These last are, influenza, dysentery, and the true plague. These and the endemics are shown to be greatly influenced by the infectious atmosphere, and in many instances to be innocuous without it. From this cause, Dr. A. conceives that Cleghorn has confounded endemics with contagions, and Sydenham contagions with endemics. One important inference is drawn from the whole, viz. that a subject under the fever from infectious atmosphere is not contagious, and that in proportion as we prevent the causes of such an atmosphere, by an improved mode of living among the various classes of society, we shall be secured from the effects of endemic effluvia, and pestilential constitutions of the air.

" Considering the events above related, and witnessing them, as Sydenham did, he may at least stand as well excused in confounding the sources of endemics, infection, and contagion, as the no less cautious Cleghorn, who boldly asserts, that " these tertian fevers have as good a right to be called infections as the small-pox, measles, or any other disease."—" For although," continues he, " there certainly is a peculiar disposition in the air to affect numbers in the same way, yet those who are much conversant with the sick are most liable to catch the distemper;" that is, is, by the infected air they breathe they become diseased, and that disease is superceded by the more powerful impression produced by the endemic miasma. Thus the only difference in the error, between these justly celebrated writers, consists in one considering all these diseases as endemics, and the other as contagions. Had Sydenham practised in a remote part of the country, where the introduction of small-pox contagion can always be traced, or had Cleghorn treated intermittents only among a scattered peasantry, instead of a camp or military hospital, probably if neither of them had been confused by the infectious atmosphere, which the seat of their practice produced, both would have avoided their error, and posterity have derived still greater advantages from their labours.

"On this account it will be absolutely necessary, as far as possible, to ascertain the laws of an atmosphere, rendered infectious by accumulating the sick, and also the changes produced in the human body by the application of such an atmosphere. In order to distinguish this, which we have called *infection*, from epidemics, endemics, and contagion, we are to consider that, though contrary to the two first, the cause is to be looked for in the human secretions, yet for its production the accumulation of numbers of sick, labouring under any complaint, is sufficient. Thus, in its origin, it differs from contagion, which can only be excited by its own particular disease. The next question will be, Whether, when once excited, it can be communicated like contagion; that is, whether the secretions of an individual, labouring under the disease excited by the infectious atmosphere, can infect another person in a similar manner; or whether the presence of such an atmosphere, or the accumulation of numbers, will be necessary to produce such an effect?

"In this enquiry we must recollect, that the actions excited in the human body by this atmosphere, are not always similar, though hitherto we have not succeeded in discovering any difference in the properties of the cause which should lead to such a difference in the effect. The mildest form in which we see disease thus excited, is a general vesicular appearance about the hands and fingers, which is contagious in a degree, in some measure dependant on the situation of the subject to whom it is communicated. If to one of the children of poverty, among whom the cause has originated, the effect is pretty similar. If to one in better condition, a few solitary vesicles will appear, and soon disappear spontaneously. But as long as the cause continues among the former, the effect will remain, varying in different subjects, according to partial exposure and other accidental circumstances; sometimes assuming the appearance of *favus*, as described by Dr. Willan, or a scabbiness about the face and scalp, as mentioned by Dr. Jenner. The latter gives the general name of herpes, or herpetic blotches, to the whole; and perhaps if we call it *herpes pauperum*, we shall find this as descriptive a term as any appearance so general will admit. I suspect pect that the moist itch, mentioned by Dr. Gillispie, is of this kind: for it is past a doubt, that a constitution engaged in such an action, may be insensible to infections and contagions. This has been well remarked by Dr. Jenner, in his vaccine experiments, and I can add my own most ample testimony to the same. Those who have had the largest opportunities, and improved them the best, describe a variety of other diseased forms which the infectious atmosphere induces. Among these are diarrhœa, dysentery, inflamed eyes, sore legs, and gangrenous ulcer. These, under an aggravation of the cause, are sometimes suddenly superseded by the worst form of infectious fever.

"In considering how far the subjects, thus affected, become contagious, we must then admit that such secretions, as can be retained in a substantial form, have the power of contagions. Not, however, with the uniformity of those well marked contagions, which may be justly called Morbid Poisons. The forms themselves are uncertain, and their effects vary not so much with the constitution of the persons exposed, as with other external circumstances. Thus all the varieties in small-pox are, as far as we can judge, independent of the degree of impregnation in the atmosphere, or concentration of contagion. Of the infectious atmosphere, on the contrary, we shall find these degrees inducing a corresponding affection in the persons exposed: in small-pox, whether we apply the contagious matter in a substantial form, or by effluvia, we produce the same disease, though in a milder degree. From infectious atmosphere the whole disease often varies with the form in which it is applied. And though we admit that these secretions, in a subtantial form, may excite similar actions in another person, yet the enquiry still remains, how far the insensible secretion or effluvia, from a person under the infectious fever, is contagious.

"It is established beyond a doubt, that the infectious atmosphere may be so attached to many things long retained in it, as to infect those who are exposed to such things, when removed from the source of the atmosphere. But if these substances, generally called *fomites*, are imbued with the atmosphere from the *crowded sick*, the property thus acquired does not prove, that the effluvia from an *individual*, infected by such fomes, would produce the same effect. To ascertain this, we must refer to certain facts, their series, and order: and as the question will not admit of common experiment, on account of the subjects affected by it, we must trace with minute diligence such records as are well authenticated, and by the frequent recurrence of uniform consequences, form our decisions, till they are contradicted by subsequent events.

"The first consideration, in these cases, is the place in which an individual is infected. If two friends are in Newgate, and one of them under a fever; should the other be seized with the same, whilst attending upon the first, no one will pretend to ascertain whether he was infected by such an attendance, or from the same cause that infected his friend. We are next to recollect, that the effect produced duced is in proportion to the degree with which the atmosphere is impregnated, or, as it is usually called, in proportion as the infection is concentrated, joined to the susceptibility of the persons exposed. Let us suppose, for instance, that the air of a prison is at first pure. The despondency and little ailments unattended to, under the general distress, vitiate this air so gradually, that the inhabitants are familiarized to it, and though none may be in high health, yet actual fever may not be perceived. A stranger is introduced from a pure air, and seized with fever, from his greater susceptibility. The air now becomes more vitiated from this greater accession of cause, and though the patient may recover, yet his recovery will not restore the air to the state in which he found it. The next person, therefore, that is admitted, is seized with greater violence, in proportion as the cause is greater, though his susceptibility may have been the same. The air is further vitiated as the same events are multiplied ; at last the jailors are infected, though accustomed to a certain degree of infection ; and strangers are seized with a suddenness and violence proportioned to the concentration of the cause, to their degree of exposure, and of their own susceptibility.

" In 1586, happened what is called a black assize at Exeter; the circumstances attending it are so remarkable, that it seems difficult in these times to conceive how the cause should have been so mistaken. A number of Portuguese sailors, taken prisoners at sea, were put into the common jail, where they remained some time before they all fell sick. The sickness soon spread through the jail, so that when the sessions arrived, the prisoners were many of them brought in hand-barrows to be tried. The mortality in the court was dreadful. But what I wish to remark is, that because the Portuguese, from being new to the prison atmosphere, were first infected, and by their numbers and consequent disease, no doubt, contributed further to vitiate the air of the prison, they are considered by Baker as having introduced an infection, which did not appear among them till they had been some days confined, without change of cloaths, in a " *deep pit and stinking dungeon.*"

" Of the black assize at Oxford, it is particularly said, that the disease and mortality, though affecting near 300 people, did not spread beyond those who were immediately exposed to its influence.

" In the black assize at Taunton, not only the court were infected, but many in the town ; which was easily accounted for, because the infected prisoners were brought from Ilchester jail, and, it is unnecessary to add, the probability that the whole populace flocked to see them on their arrival.

" The last black sessions in the Old Bailey was held on the 11th of May, 1750. On the 13th, died Alderman Lambert; on the 14th, R. Cox, under sheriff; on the 17th, Baron Clark; on the 19th, Sir T. Abney, Justice of Common Pleas, T. Otway, barrister, W. Baird, ditto, W. Sharplop, and four others; on the 20th, the Mayor and eight of the Middlesex jury. All these individuals were were seated in the course of an atmosphere likely to pass from the prisoners to an open window. The rest of the court escaped.

"At the assizes at Oxford, it is evident, by the history, that no one was infected but those who were in court, for their sickness was by some imputed to causes whith could only influence such as were present. In London, too, there is reason to believe that no persons were affected in the families of the deceased; because the list of deaths in the Gentleman's Magazine, which is very precise in marking all who died from this event, gives no account of any but such as were in the court; nor do the subsequent remarks on the calamity hint at any further extension of the infection. But we are not on that account to determine that such a fomes is, under every circumstance, innocuous beyond its immediate source, since we find, in two years after, a fever from the same jail was communicated to the families of the infected."

The manner in which the disease afterwards spread in some instances, and in others ceased with the persons first infected, is next traced; and the author having thus ascertained, with as much exactness as the subject will admit, the laws of this atmosphere, proceeds to show its influence on the endemics and epidemics. This produces an important inquiry into the Yellow Fever and Plague, the contagious properties of which are said not to be ascertained, and the causes of the disagreement among authors on this subject are minutely investigated. This part of the work is too important to be abridged, we can therefore only recommend it to the careful perusal of all those who are likely to be engaged practically in these formidable diseases.

The 3d chapter is on the Exanthemata; of this the small-pox, as the most formidable, and comprehending the terrors of all the rest, is most minutely described, and with the mode of cure is comprehended the treatment of all other acute fevers, the early symptoms and sources of danger being similar in most of them.

The last chapter, On Prevention, comprehends the various and prophylactic means for each class of diseases above enumerated. Inoculation, Variolous, and Vaccine, are candidly discussed; and whilst the author makes no difficulty concerning the security of the latter, he deprecates all restrictions on the former, till the public mind is better reconciled to so extraordinary a revolution. This, however, relates only to the metropolis, in which the disease is said to be always more or less epidemic.

Such is the nature of this work, of which, from the quantity of information it contains, we can give only an imperfect sketch. To recommend it to our readers would be superfluous, as the importance of the subject, and the abilities of the author, in this particular branch of the science, are both well known to the medical world; we shall just remark, that, besides the quantity of other useful matters contained in the work, we feel highly obliged by the perspicuity with which three subjects are treated, each of which have so much divided the writers of the present day, as to convince us that that they have not clearly understood each other.—The language and theories of Mr. Hunter; the true discrimination of the venereal disease, and such as have been confounded with it; and the precise history and character of the vaccine vesicle.

